# Superficial punctate keratopathy in a pediatric patient was related to adenoid hypertrophy and obstructive sleep apnea syndrome: a case report

**DOI:** 10.1186/s12886-018-0720-7

**Published:** 2018-02-23

**Authors:** Ying-ying Gao, Hong-juan Wang, You Wu

**Affiliations:** Department of Ophthalmology, the Second Affiliated Hospital, Fujian Medical University, Quanzhou, Fujian 362000 China

**Keywords:** Superficial punctate keratopathy, Floppy eyelid syndrome, Obstructive sleep apnea

## Abstract

**Background:**

Known causes of superficial punctuate keratopathy (SPK) in children include entropion, viral infection, blepharokeratoconjunctivitis (BKC), and toxicity of eye drops. However, there are some SPK patients whose causes could not be identified well. Herein, we describe the history, diagnosis, treatment, and prognosis of a rare case.

**Case presentation:**

To report a case of superficial punctate keratopathy (SPK) which coexisted with floppy eyelid syndrome (FES) and presented as intermittent red eye and blurred vision in an 11-year-old boy who slept in the prone position. His condition did not improve despite treatment with topical antibiotics (levofloxacin, tobramycin), steroid eye drops (prednisolone), and artificial tears. The patient was diagnosed with tonsil hypertrophy and nasopharyngeal adenoid hypertrophy and obstructive sleep apnea syndrome (OSAS). He underwent tonsillectomy and adenoidectomy. Then he started sleeping in the supine position postoperatively. The SPK, red eye and blurred vision completely resolved after surgery without additional treatment. The corneal sensation also recovered gradually during the next 7 years. However, the floppy eyelid did not resolve.

**Conclusion:**

Recurrent SPK of childhood might be related to tonsil hypertrophy, adenoid hypertrophy and OSAS, which can be rehabilitated by a surgical approach.

## Background

Known causes of superficial punctate keratopathy (SPK) in children include entropion, keratoconjunctivitis, and eye-drop toxicity [1–3]. However, there are some cases of SPK where the cause is not clearly identified. Here, we describe a case of childhood SPK that coexisted with floppy eyelid syndrome (FES) and presented as intermittent red eye and blurred vision which was diagnosed with tonsil hypertrophy and nasopharyngeal adenoid hypertrophy and rehabilitated by a surgical approach.

## Case presentation

A 11-year-old boy with complaints of intermittent redness and blurred vision in the right eye for 2 years was referred to us in September 2009. The redness and blurred vision typically occurred in the morning once every 2–3 days and gradually subsided an hour later. His vision markedly worsened, such that he could not even identify an acquaintance from 5 m away if his left eye was covered. His condition did not improve despite multiple consultations with different ophthalmologists and several treatments with topical antibiotics (levofloxacin, tobramycin), steroid eye drops (prednisolone), and artificial tears. Punctate corneal keratopathy existed in the right eye at each visit according to previous medical records. His history included allergic rhinitis that presented as stuffy nose and frequent sneezing, for which no appropriate treatment was sought. Upon inquiring about the patient’s sleeping habit, his parents informed us that he usually slept in a prone position tilted to the right side and snored at night.

**Fig. 1:**
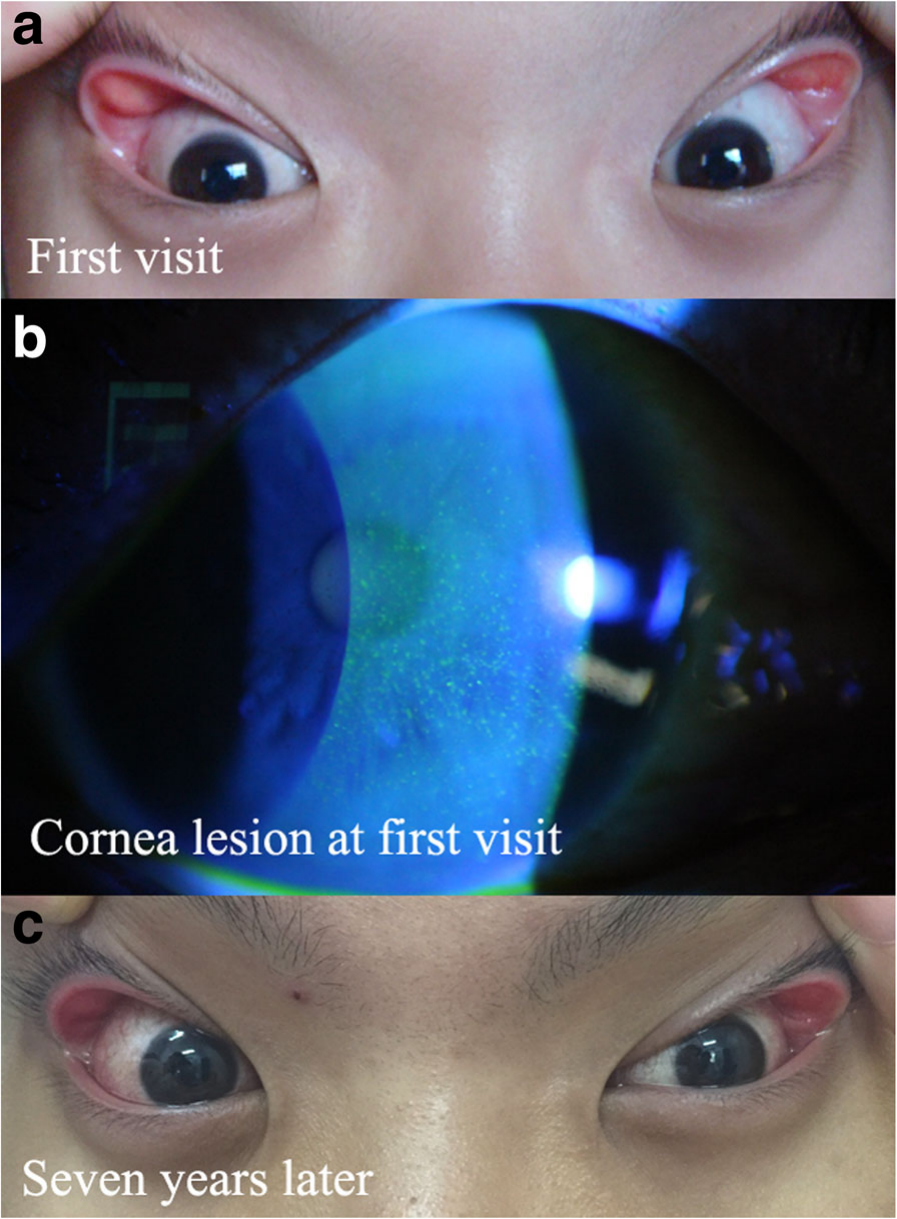
Grade-2 floppy eyelids and mild bulbar conjunctival hyperaemia were noted in both eyes at the first visit (**a**). Scattered, fine, punctate corneal epithelial damage was confirmed by fluorescein staining of the right eye (**b**). The degree of floppy eyelid remained the same 7 years later (**c**)

An ocular examination revealed that the uncorrected visual acuity was (20/60), and his best-corrected visual acuity was (20/20) in both eyes in the afternoon. The patient had grade-2 floppy eyelids in both eyes (Fig. 1a) [4]. Under slit-lamp examination, the eyelid was in the right position, the bulbar conjunctiva was mild hyperemic at both side; and papillary hyperplasia was noticed in both the upper tarsal conjunctiva, with the right eye more severe than the left. Scattered, fine, punctate corneal epithelial damage was confirmed by fluorescein staining on the right eye (Fig. 1b). Corneal sensation was 25 mm in the right eye and 50 mm in the left eye as measured using a Cochet-Bonnet esthesiometer. No other abnormity was detected in either eye.

Considering that the floppy eyelid might be related to OSAS [5, 6], the patient was subsequently referred to the Respiratory Department and moderate OSAS was confirmed by overnight polysomnography. Computed tomography showed severe tonsil hypertrophy and nasopharyngeal adenoid hypertrophy. The child was then referred to an ear-nose-throat (ENT) specialist, and a combined operation (tonsillectomy and adenoidectomy) was performed 2 days later.

The redness and blurry vision in the right eye disappeared after surgery without other additional topical eye drops. Eye redness happened 4 times without vision involvement during the 7-year follow-up duration. Corneal sensation gradually returned to normal: 45 mm 1 year later, 45 mm 3 years later, 60 mm 7 years later. The patient seldom slept in the prone position and corneal lesion disappeared thereafter. The corneal sensation returned to normal (60 mm) in both eyes at the last follow-up. However, the degree of floppy eyelid remained the same (Fig. 1c). After 7 years the child’s corneal diopter is still in the normal range and there is no tendency of keratoconus.

## Discussion

OSAS has been associated with numerous ophthalmological disorders, including floppy eyelid syndrome, visual field defects, retinal vein occlusion, central serous chorioretinopathy, and certain optic nerve dysfunctions, such as papilledema, non-arteritic anterior ischemic optic neuropathy, glaucoma, and decreased peripapillary nasal retinal nerve fiber layer (RNFL) thickness [7–11].

It is known that OSAS leads to FES because chronic hypoxia at night leads to degradation of elastin and collagen in the eyelid [5, 6]. The association between FES and OSAS is well known, as is that between FES and keratopathy. However, FES, OSAS and related keratopathy are reported to happen in obese adults who snore at night [12–14] but rarely seen in pediatric patients. The unusual complaints, clinical findings and its association with rhinopharyngeal abnormalities in this case were easily neglected by both ophthalmologists and pediatricians.

It has been reported that chronic rhinitis in children is related to adenoid hypertrophy, thereby causing upper airway obstruction leading to OSAS, especially at night [15, 16]. When the patient was in the supine position the retro-displacement of the tongue root worsened the upper airway obstruction and hence, the child resorted to the prone position during sleeping. As a result of the easy upper eyelid eversion in FES, the right-sided prone position caused the right eyelid to move away from the eyeball, leading to dryness of the ocular surface and subsequent corneal lesion and edema in the morning that manifested as redness and blurred vision. Once the patient was awake, the eyelid returned to the normal position, the conjunctival congestion disappeared, and the corneal edema resolved. Subsequently, the patient’s vision became clearer and the corneal lesion also decreased over time. However, the punctate lesion remained. The severity of the symptoms depended on the sleeping conditions and presented intermittently over the week.

In our patient, the corneal sensation was 25 mm in the right eye and 50 mm in the left. The dull sensation in the right cornea could not stimulate the trigeminal nerve, which would otherwise have caused discomfort and sent appropriate signals to the central nervous system so that the body position could be changed to prevent eyelid malposition and further damage the cornea. The cause of decreased corneal sensation in the right eye might have been related to the chronic nocturnal hypoxia and recurrent corneal erosion, although this theory still needs to be proved.

After tonsillectomy and adenoidectomy, there was no airway obstruction during sleep, and the child did not require to resort to the prone position. After this malposition was rectified, the symptoms and corneal lesion disappeared; The corneal sensation returned to normal at 7-year follow-up period. Interestingly, our patient’s scores at school markedly increased postoperatively, which are likely to be a direct consequence of eliminating the nocturnal hypoxia and thereby improving his sleep quality, which positively influenced his daytime learning efficiency.

After the consultation for this case, we also found children with SPK caused by OSA during the next 7 years; however, their symptoms and signs were not so typical and detailed. Herein, we report this rare case to provide a reference for clinical doctors.

## Conclusion

Our case reinforces the fact that ophthalmologists should pay close attention to the sleeping habits, floppy eyelid degree and any nasopharyngeal abnormalities in children that may be linked to OSAS in cases of refractory corneal lesion. Such patients can be rehabilitated by a surgical approach.

## Abbreviations

BKC: Blepharokeratoconjunctivitis
ENT: Ear-nose-throat
FES: Floppy eyelid syndrome
OSAS: Obstructive sleep apnea syndrome
SPK: Superficial punctate keratopathy

## References

[1] Skorin L (2003). A review of entropion and its management. Cont Lens Anterior Eye.

[2] Bernauer W, Langenauer UM (2006). Chronic (kerato-) conjunctivitis refractory to therapy in children. Klin Monatsbl Augenheilkd.

[3] Kanamoto T, Kiuchi Y, Tanito M (2015). Comparison of the toxicity profile of benzalkonium chloride-preserved tafluprost and SofZia-preserved travoprost applied to the ocular surface. J Ocul Pharmacol Ther.

[4] Liu DT, Di Pascuale MA, Sawai J (2005). Tear film dynamics in floppy eyelid syndrome. Invest Ophthalmol Vis Sci.

[5] Netland PA, Sugrue SP, Albert DM (1994). Histopathologic features of the floppy eyelid syndrome. Involvement of tarsal elastin. Opthalmology.

[6] Schlotzer-Schrehardt U, Stojkovic M, Hofmann-Rummelt C (2005). The Pathogennesis of floppy eyelid syndrome:involvement of matrix metalloproteinases in elastic fiber degradation. Ophthalmology.

[7] Leroux les Jardins G, Glacet-Bernard A, Lasry S, Housset B, Coscas G, Soubrane G (2009). Retinal vein occlusion and obstructive sleep apnea syndrome. J Fr Ophtalmol.

[8] Jain AK, Kaines A, Schwartz S (2010). Bilateral central serous chorioretinopathy resolving rapidly with treatment for obstructive sleep apnea. Graefes Arch Clin Exp Ophthalmol.

[9] Hayreh SS, Zimmerman MB, Podhajsky P, Alward WL (1994). Nocturnal arterial hypotension and its role in optic nerve head and ocular ischemic disorders. Am J Ophthalmol.

[10] Mojon DS, Hess CW, Goldblum D, Fleischhauer J, Koerner F, Bassetti C, Mathis J (1999). High prevalence of glaucoma in patients with sleep apnea syndrome. Ophthalmology.

[11] Casas P, Ascaso FJ, Vicente E, Tejero-Garcés G, Adiego MI, Cristóbal JA (2013). Retinal and optic nerve evaluation by optical coherence tomography in adults with obstructive sleep apnea-hypopnea syndrome (OSAHS). Graefes Arch Clin Exp Ophthalmol.

[12] Bilenchi R, Poggiali S, Pisani C (2004). Floppy eyelid syndrome associated with obstructive sleep apnoea. Br J Dermatol.

[13] Culbertson WW, Ostler HB (1981). The floppy eyelid syndrome. Am J Ophthalmol.

[14] Leibovitch I, Selva D (2006). Floppy eyelid syndrome: clinical features and the association with obstructive sleep apnea. Sleep Med.

[15] Tezer MS, Karanfil A, Aktas D (2005). Association between adenoidal-nasopharyngeal ratio and right ventricular diastolic functions in children with adenoid hypertrophy causing upper airway obstruction. Int J Pediatr Otorhinolaryngol.

[16] Quaranta N, Milella C, Iannuzzi L (2013). A study of the role of different forms of chronic rhinitis in the development of otitis media with effusion in children affected by adenoid hypertrophy. Int J Pediatr Otorhinolaryngol.

